# Selections

**Published:** 1817-02

**Authors:** 


### PART III.

### SELECTIONS.

—— Factorum est copia nobis.

### FOREIGN JOURNALS.

PHYSIOLOGY.—*Memoir on the First Inspiration of the new-born Child.*

( Continued from page 68. )

IF the shock of the diaphragm be the constant effect of compression of the thorax in the dead subject, what produces this compression on the living muscle? Doubtless, the shock received acts upon it as a stimulus, exciting it to contraction, the more powerfully in proportion to the force of the momentary distension of its fibres. Thus, for example, the heart is universally acknowledged to contract more forcibly, as its cavities are more fully distended with blood; and the urinary bladder and rectum contract from the mere mechanical effect of distension by urine, and fæces or injected fluids. The vital action of the intestine is excited, rather by the suddenness of distension, than by the irritating quality of the liquid introduced. Hence, when, in reducing a fracture or dislocation, the muscles are not placed in perfect relaxation, they contract violently, and render reduction impracticable: for distension of a muscular fibre acts upon it as a real stimulus, inciting it to contraction. This fact, as it regards the diaphragm, is strikingly illustrated by the following experiments:—I opened the abdomen of several puppies, previously destroyed by puncture of the spinal marrow near the head; and, having compressed the inferior part of the thorax, and suddenly removed the compressing force, I saw that the diaphragm experienced, at first, a twitching, which, afterwards, terminated in contraction. At other times, when the irritability of the animal was nearly extinct, the fibres of the diaphragm were yet affected by a sort of vital tremor, sufficiently indicating the susceptibility of the muscle to the stimulus which resulted from compression of the ribs. If, on the other hand, the animal were yet living, its inspiration was evidently increased; as Bichat has observed, on forcibly and suddenly compressing the thorax of animals already weakened by hæmorrhage. It is generally known, that slight irritation suffices to provoke contraction of the diaphragm; which, next to the heart, is the most irritable of muscles, and preserves longest after death its vital force. The intercostal muscles also stand high in the scale of irritability, and their fibres become equally tense from compression of the thorax; which, in depressing the ribs, enlarges the intercostal spaces. Even the muscles of the fœtal extremities contract with force, on an attempt to extend them, as is well known by all who have performed the operation of turning the child *in utero*. The foregoing experiments on the diaphragm I have not repeated in adult animals; but, doubtless, the results would be similar.

Now, if compression of the thorax excite the action of the diaphragm, what circumstance would more effectually induce this action, than the constriction suffered by the thorax of the fœtus in its passage from the pelvis? This part of the child is certainly smaller in every direction than any of the straights of the pelvis; but the considerable increase of bulk from the application of the arms to the chest should be considered, and the diminution of the antico-posterior diameter of the inferior pelvic aperture by restoration of the coccyx, after the passage of the head. Moreover, without calculating the capacity of the pelvis, the resistance of the soft parts, as the uterine orifice, vagina, and vulva, suffices, from their elasticity and vital properties, to produce on the fœtus the effect in question. In footling cases, the inferior limbs of the child will be agitated, from the compression exercised on the trunk or buttocks by the soft parts of the mother: and, provided the trunk be rapidly expelled after the shoulders have passed, the instantaneous but strong compression of the thorax suddenly ceases; and the ribs, in rebounding, produce that motion of the diaphragm which directly incites this muscle to contraction.

This, then, is the principal agent of respiration, excited by the very act of delivery; and the most difficult point of the problem is thus solved. Hence, it is clear, why respiration coincides with birth; and, to explain this phænomenon, we need no longer admit the exercise of the function in the unborn fœtus.

Expiration is generally attributed to relaxation of the inspiratory powers, and considered as a return from action to repose. Yet the lungs, I think, are not absolutely passive in this second act of respiration; but that, stimulated by the air, they re-act from their tonicity, and thus contribute to partial expulsion of the inspired fluid. I am even inclined to regard the dilatation of the pulmonary cells, as effected by a sort of action in the organ, enabling it to expand during inspiration. Yet this action of the lungs invariably accords with the motions of the thorax: otherwise, there would result a space between the two pleuræ, never naturally existing.

Inspiration again follows expiration: and here a new question presents itself, with respect to the alternation of the respiratory movements; which, as foreign to my subject, I can only answer by observing, that I believe the second inspiration to depend on volition of the infant, excited by the restraint and uneasiness experienced by it when the state of expiration is unduly protracted: for, if volition influence not the exercise of this function, why has it been subjected, by nature, to the empire of the will? To this position, I am aware, may be opposed arguments drawn from the state of sleep or disease, during which respiration goes on freely, although the influence of volition is suspended. But is not the stimulus of volition, under many circumstances, replaced by the re-action of the brain, excited by the movements which radiate from the internal organs? Thus the locomotion of somnambulists, the exercise of the voice in disturbed sleep or delirium, and the involuntary contractions of the muscles in convulsive diseases, sufficiently prove that the organs subjected to volition, may become independent on it. The motions of the intra-uterine fœtus are, as Bichat remarks, not the effect of volition; but result from the sympathetic influence of the internal organs on the brain. In like manner, the painful sense of restraint, experienced when respiration is suspended, becomes, I conceive, a stimulus to the brain, and causes it to excite contractions of the diaphragm by the medium of the nerves, even during sleep. The same happens in the new-born child. Thus, although the action of the pulmonary organ belongs to the functions of vegetative life, and is excluded from the sphere of animal life, yet the brain has perception of the slightest derangement occurring in this organ, and acts in consequence: whence it results that the thoracic movements, although subjected to volition, seem sometimes to re-enter the class of motions of organic life, without ceasing, for a moment, to depend upon the brain. But we will now return, and answer the objections which may be thought to invalidate our theory of the first inspiration.

I. This theory, it may be urged, applies exclusively to man and quadrupeds, whose young, with difficulty expelled, are compressed by the genital parts of the mother; and not to other animals, as birds and amphibia, which, produced from an ovum, respire like the human subject. With regard to the first of these animals, although they have a double respiration like the mammalia, the organization of their thorax and lungs is widely different. They have no diaphragm, but are furnished with abdominal cells, which are a continuation of the lungs; and the latter, adherent to the ribs, are, in some wise, prolonged into the interior of the bones, the cells of which are never in a state of collapse. Thus the respiratory organs of these animals are constantly expanded, from their formation. Previously to having broken the shell, the chick is in relation with the air contained in the large end of the egg, the volume of which increases with the progress of incubation. The nostrils and trachea being permanently open at the moment of the exit of the chick from the ovum, and the bronchial ramifications and cells expanded by their adhesion to the bones, the atmospheric air rushes in as into a vacuum, and the action of the abdominal muscles alone suffices to keep up its flux and reflux in the abdominal vesicle. In brief, the main poiut, in the fœtus of man and quadrupeds, is to produce dilatation of the thorax, and arouse the lungs from their original state of collapse; while, in birds, this is already achieved by their peculiarity of organization.

Amphibia differ yet farther from the human structure. Destitute of a diaphragm, and capable of suspending, for some time, their respiration, and even of surviving the destruction of their lungs, these animals must not be brought into a parallel with man, in examining the mechanical phænomena of respiration.

II. Again, it may be asked how my opinion is reconcileable with Wrisberg's observations; according to which the thorax is moved without inspiration being accomplished? and if, on the other hand, the action of the diaphragm, excited by an external irritation, is the sole condition under which respiration may be established, why have not children, perishing in delivery, respired, although no motions have been executed by the thorax, in the first moment of birth? To these questions I reply, that movements, performed by the thorax, do not invariably imply participation of the diaphragm. The thorax may be bent in various directions, by the dorsal and vertebral muscles, without calling into action those of respiration. But, even admitting the alternate contraction and relaxation of the diaphragm and intercostals in the still-born fœtus, the circumstance, far from exploding our theory, would tend only to confirm it: proving that, vitality not yet extinct in these muscles, they have been capable of excitement by the pressure which the ribs have sustained; but that the lungs could not co-operate, either in consequence of the loss of the vital principle, or of the obstruction of the air in its entrance into the viscus by some mechanical cause, as accumulation of mucus in the trachea, or tumefaction of the laryngeal membrane. I am surprised that this remark has not been long ago made, since the state of the mucous membranes, last described, appears to be the most frequent cause of the death of the fœtus, whenever the head has remained long in the passage, and the neck has been tightly encircled, either by the umbilical cord, or the contracted orifice of the womb. In fact, when the body of the fœtus, thus destroyed, is examined, we find the cellular membrane of the neck, and the thyroid gland tumefied, the muscles, and the coats of the blood-vessels, red and minutely injected; and, particularly, the mucous membrane investing the arytenoid cartilages and ligaments of the glottis, so swollen as utterly to intercept the passage of the air. In vain will the diaphragm and intercostals then contract, to enlarge the capacity of the chest; inspiration cannot take place. In indicating the death of the lungs, as the cause of the non-introduction of air into them, even when the inspiratory muscles contract, I may seem to contradict what I have before said of the passive state of the lungs in respiration: hence some explanation is necessary. I admit the passive state of the lungs, relatively to the power, formerly attributed to them, of pumping in the atmospheric air by a force peculiar to them, and analogous to that by which the lymphatics absorb a fluid presented to their orifices. This faculty I, with all modern physiologists, deny; but I attribute to the pulmonary texture a tendency to dilate and augment the capacity of its cells, which tendency acts upon dilatation of the thorax; and I consider the development of the lungs as dependent on an internal force, similar to that possessed by the cellular structure in some parts, and manifested in the phænomena of vital turgescence. If it were otherwise, and the expansion of the lungs, in inspiration, were merely passive, one might make a corpse inspire, and even resuscitate the dead, provided the thoracic parietes were artificially dilated. Now it is this defect of the vitality of the lungs, by virtue of which they answer to the dilatation of the thorax, which takes place in still-born children, as mentioned by Rœderer; and this, again, is one of the causes why children do not respire, although their their thoracic muscles have contracted.

(From the unusual length of this Memoir, and our wish to render a full account of it, we are reluctantly induced to defer its conclusion till March. Editors.)

---

### *On the Intestinal Gases of the Human Subject in Health.**

M. Jurine, of Geneva, is, I believe, the only one who has yet analysed these gases. In a Memoir, which gained a prize from

---

* Note sur les Gaz intestinaux de l'Homme Sain, par M. Magendie.—Annales de Chimie et de Physique; Juillet, 1816.

the Medical Society of Paris, in 1789, he delivered the results of some experiments, made on the body of a lunatic who had perished from cold in his cell, and was immediately opened. He discovered, in the intestinal canal, oxygen gas, carbonic acid, nitrogen, and sulphuretted hydrogen; and, moreover, established, that the proportion of carbonic acid was greater, both in the stomach and small intestine, than in the large one; while that of the nitrogen was in an inverse ratio. But, at the period of these experiments, the eudiometric apparatus was very imperfect; and, besides, they were made on one body only. Since then, the eudiometer has been brought to great perfection, and the chemist and physiologist are become much more rigid in their researches: hence these experiments leave much to be desired.

Having at my disposal, in the years 1814 and 1815, the bodies of four persons recently executed, I thought that it might be useful to resume an investigation, which, from the very period at which it was begun, must necessarily be imperfect. M. Chevreul had the kindness to assist me in the analysis.

At Paris, the condemned commonly take, an hour or two before execution, a light repast: thus their digestion is in full activity at the moment of death.

In collecting the different gases, I employed means calculated to prevent the mixture of those of the stomach with those of the small intestines, and of the latter with those of the large. They were all collected under mercury, a precaution not taken by M. Jurine, and which must necessarily have influenced his results, several of the intestinal gases being soluble in water. M. Chevreul and myself endeavoured, in our first experiments, to determine the nature of the gases contained in the three portions of the intestinal canal. We found in the stomach oxygen gas, carbonic acid, pure hydrogen, and nitrogen; in the small intestine, the same, with the exception of oxygen; in the large intestine, carbonic acid, nitrogen, carburetted hydrogen, and sulphuretted hydrogen.

The nature of the different gases determined, we wished to ascertain precisely their relative proportions, and obtained the following results:

| Exp. I. Male, aged 24, took, two hours before execution, prison bread, cheese, wine and water. | | Exp. II. Male, aged 23; same food, same interval. | Exp. III. Male, aged 28, took, four hours before execution, bread, boiled beef, lentils, and red wine. |
|---|---|---|---|
| Stomach contained, | | | |
| Oxygen gas — | 11,00 | Merely a bubble of gas, incapable of analysis. | Not analysed. |
| Carbonic acid gas | 14,00 | | |
| Pure hydrogen gas | 3,55 | | |
| Nitrogen gas — | 71,45 | | |
| Total | 100,00 | | |

| Exp. I. continued. | | Exp. II. continued. | | Exp. III. continued. | | |
|---|---|---|---|---|---|---|
| Small Intestines contained, | | | | | | |
| Carbonic acid gas | 24,39 | — — | 40,00 | — — | | 25,00 |
| Pure hydrogen gas | 55,53 | — — | 51,15 | — — | | 8,40 |
| Nitrogen gas — | 20,08 | — — | 8,85 | — — | | 66,60 |
| Total | 100,00 | — — | 100,00 | — — | | 100,00 |
| Large Intestines contained, | | | | | Cæcum. | Rectum. |
| Carbonic acid gas | 43,50 | — — | 70,00 | — | 12,50 | 42,86 |
| Carburetted hydrogen, | | | | Pure hydr. | 7,50 | |
| with sulphuretted hydrogen gas — | 5,47 | Pure and carburetted hydrogen — — | 4,60 | Carburetted gas — | 12,50 | 11,18 |
| Nitrogen gas — | 51,03 | — — | 18,40 | — | 67,50 | 45,96 |
| Total | 100,00 | — — | 100,00 | — | 100,00 | 100,00 |

Some traces of sulphuretted hydrogen were evident on the mercury before the experiment.* These results may be depended on, for nothing was neglected that could insure their accuracy. They accord pretty nearly with those long since obtained by M. Jurine, as regards the nature of the gases; but they invalidate the statements of this gentleman respecting the proportion of carbonic acid, which, according to him, regularly decreased from the stomach to the rectum. We have just seen, on the contrary, that this gas is, in general, more abundant in the large intestine, than in the small one, or the stomach.

### Pathology. *Case of Chronic Inflammation of the Brain, which became acute a short Time before Death.*†

N. C. aged 12, a boy of lymphatic temperament, indolent, but possessed of quickness and a strong memory, studied in the Lyceum of Paris. In November, 1814, he complained of pain in the right suborbitar region; but his appetite continued. His masters, thinking him idle, spurred him to exertion. This state

* The preceding Table, which, for brevity's sake, we have constructed from the materials of M. Magendie, will be readily understood. In the third experiment, the gases of the cæcum and rectum were separately analysed. From the numeration of the experiments in the original, it should seem that the author had made them on all the four bodies; but, by some mistake, he has omitted to record the results of the second.—Editors.

† Observation sur une Phlegmasie lente du Cerveau, &c. par M. Leon-Caigné, D. M. à Neuilly. Journal de Médecine, &c. par Leroux, Mai. 1816.

lasted eight or ten days, and the boy resumed his lessons as usual. About the middle of the ensuing January, the pain became more acute; and his appetite and cheerfulness disappeared. He was then brought to Neuilly. Eight days' rest removed the pain, and restored his wonted cheerfulness. He, himself, asked to be sent back to the Lyceum. Fifteen days after his return, same pain; variable appetite; depression; and neglect of his duties, again ascribed to indolence: hence he was menaced with punishment. These symptoms continued for some days. The boy no longer ate or played, and constantly held his head between his hands. He was again sent to Neuilly. On his arrival, his countenance was decomposed; his pulse small and contracted; suborbitar pain intense; tongue red and moist; a deep inspiration, every six minutes; bowels regular. *Prescribed a nitrous potion and simple enema.* 3d day. No sleep during the night; inspiration plaintive; eyes fixed. 4th. Suborbitar pain more intense; the hand constantly directed to the forehead; eyes fixed: at ten o'clock, convulsions, and rigidity of the limbs, continuing half an hour, and succeeded by screams; the boy thinking himself about to fall down a precipice, and calling for help. *Pediluvia, leeches, sinapisms to the feet.* Fourteen hours afterwards, the same convulsions, and terror at the close of the crisis. *Leeches repeated; blisters to the nucha and legs; nitre whey; and sedative potion.* Night tranquil, but sleepless.—6th. Same pain; eyes constantly fixed; pulse slow; answers given to every question; vision double. The boy asked for bread and sweetmeat, which he greedily swallowed. *Potion repeated.* The blisters have acted well: night calm. —No alteration till the 12th. Sub-orbitar pain violent; occasional screams. Convulsions towards evening. *Pediluvia; ethereal drops; both sides of the head shaven and blistered.* On the decline of the crisis, vision constantly double: the right eye more open than the left, which remained fixed. *Remedies continued.*—14th. Nervous cough; deglutition difficult; sub orbitar pain severe.—15th. Cough increased; deglutition more difficult.—16th. The boy can no longer swallow without dread of suffocation; respiration stertorous.—Evening. Can no longer take any thing; tosses from side to side: inspiration rattling. He thrust his fingers into the fauces, in the hope of removing the obstruction. Night restless. Died next morning.

*Dissection.* The membranes of the brain were found injected with blood; the longitudinal sinus completely filled with black blood; all the other vessels exceedingly gorged. A mass of black and coagulated blood was seen in the anterior part of the brain, between the membranes, on a level with the right frontal fossa. On compressing the anterior lobes of the brain, much blackish and fluid blood oozed from its fissure. The lateral ventricles contained about four ounces of reddish serum. The cerebellum was in an advanced state of inflammation, and its membrane injected throughout. The whole cerebral mass was of considerable volume; its cortical substance diseased in the anterior, and several other parts.

*Reflections.* It seems certain, that a point of irritation commenced in the cerebral membranes of the right side, about the frontal fossa; that a spontaneous congestion of blood had there taken place; and, consequently, determined membranous inflammation; which, becoming active, induced general disorder of the whole mass: and, lastly, that the double vision was caused by pressure of a coagulum of blood on the optic nerve.

---

### *Memoir on Inflammation of the Hernial Sac.**

Few surgical diseases claim more attention than hernia; whether we consider its danger or the delicate operations which it requires. I speak not of those barbarous and useless operations for its radical cure, which an enlightened practice has long proscribed. Few diseases are as well understood. On every point relating to the anatomy and treatment of hernia, dissection, aided by clinical observation and experience, has thrown the strongest light.

Yet one circumstance seems hitherto to have eluded observation. At least, I have not found it recorded in any writings upon hernia. But having thrice, within a short time, observed it, I presume that it is not rare. That to which I allude, is inflammation, chronic or acute, of the hernial sac, with congestion and morbid thickening of the parietes, as existing without actual displacement. All writers on the subject mention thickening of the parietes of the sac in old hernia. But they indicate it as merely occurring in strangulated herniæ, and then requiring some variations and precautions in the operative process. Probably, the defect of observation on this morbid state may be explained from its exciting no prominent symptom, or simulating other diseases; and hence being concealed by the patient, or mistaken by the practitioner

Inflammation and thickening of the parietes of the hernial sac give rise to a tumour, not of itself commonly dangerous; but which, assuming the character of other affections, or complicated with them, may lead to dangerous or fatal mistakes. Hence I have considered it right to claim the attention of surgeons to this subject, and communicate the observations and reflections which I have made upon it. In the first place, I shall trace the history of the disease, comprehending its causes, characters, progress, and termination. Subsequently, I shall enumerate the diseases which it simulates, or with which it may be confounded; and deliver the diagnostic signs, proper to obviate mistake. Lastly, I shall say something on the treatment of these cases.

---

* Memoire sur la phlegmasie du sac herniaire, par M. Duparque, D. M. Bulletin de l'Athénée de Médecine de Paris, Juin, 1816.

*First Case.* Rituy, a baker, aged 75, and of strong constitution, became, from his age and infirmities, an inmate of the *hospice de Bicêtre.* For twenty years he had carried two inguinal herniæ, the consequence of violent muscular exertion. They were readily reduced, on their first appearance, and confined by a double elastic truss. This, however, did not prevent them from often being, one or both, displaced: yet the patient was always able to return them himself. A slight effort, on the 1st of July, 1811, caused both herniæ to come down, and the patient, after a vain attempt to put them up, re-applied his truss; but symptoms of strangulation obliged him to enter the infirmary five days afterwards. At that time, the left tumour, very large, descended into the scrotum. It was soft and indolent. I could clearly distinguish in it, by the touch, a loop of intestine distended by gas and fæces. The right tumour was much less voluminous, harder, glistening, and painful. The patient said that his pains had only come on since the truss was re-placed. Obstinate constipation; swelling and sensibility of the abdomen, especially in the vicinity of the inguinal rings; continual eructation; nausea; vomiting, at first of partly-digested aliment, and afterwards of fœcal matter, were symptoms evidently dependent on mechanical obstruction of the intestinal tube; and consequently indicated the existence of strangulation. To these symptoms were joined urgent thirst, extreme anxiety, and a frequent and quick pulse.

Aperient potions, and injections made laxative by the addition of sulphate of soda, emollient cataplasms to the abdomen and tumours; the warm bath, and cautious employment of the taxis, readily affected the reduction of the left hernia: but the right, although smallest, resisted these means. On the morning after, I found it in the same state; and yet one of my colleagues assured me that he had succeeded in returning it; but that, notwithstanding the application of a truss, it had shortly re-appeared, and could not again be put up. I, however, by fresh trials, got nearly the whole of the tumour beyond the ring; but, on removing the pressure, it was instantly protruded anew. In this way, probably, the reduction had been before effected by my colleague. From the dilatation of the ring, I considered that the strangulation was caused by the neck of the hernial sac. The tumour, large as a hen's egg, was nearly pyriform, and prolonged by its summit into the ring; to the bottom of which it seemed to adhere. It was hard, glistening, and painful on pressure. Yet the patient referred his worst pains immediately behind the rings, particularly that corresponding to the tumour.

The symptoms grew worse: and acute inflammation, seizing the tumour, was propagated to the integuments. The failure of every attempt at reduction, united to the deplorable state of the patient, indicated the operation as our last resource. It was performed on the ninth day of strangulation.

On exposing the tumour in the usual mode, a smooth and highly red surface came into view; traversed, in the middle, by a superficial longitudinal groove, which seemed to indicate the separation of the two portions of a loop of intestine. All the cellular membrane was removed, and yet no sac visible. The resistance of the tumour prevented its elevation by the pincers. It now became a question what were the contents of the sac, whether omentum or intestine, or both, intimately connected to each other, and to the sac, by adhesions, consequent on the existing inflammation? In this dilemma, with a view of preserving important parts from injury, the ring was dilated, and an attempt made to reduce the tumour altogether, but in vain. An obstacle, situated behind the ring, rendered it impracticable. The tumour, nearly returned, again suddenly came down, on discontinuing the pressure. The resistance, seemingly caused by adhesion, was plainly distinguished by the finger; but it was too firm to be destroyed. Indeed, the operator was restrained by fear of aggravating the mischief, and prudently resolved to leave all to nature.

I now most attentively examined the tumour: it was smooth, of an obscurely red colour, like that of inflamed intestine; but this colour was merely superficial, and depended on minute injection of the capillaries of a very thin cellular layer, intimately adherent to it. Where this layer had been removed by the knife, the tumour was of an almost dead white colour. Its parietes sank, on pressure, with a crepitous sound; but soon rose again, on removal of the pressure. Its summit, more thin and soft towards the ring, entered that orifice, and became confounded with the resistance which, as before mentioned, opposed its return.

The alarming symptoms, which seemed to menace approaching death, spontaneously subsided, to our great astonishment, after two days. Stools abundant; cessation of vomiting; tension, hardness, and pain of the abdomen diminished. The tumour remained unaltered.

July 12th. Our patient, feeling tolerably well, ate some kidney-beans; and, from that moment, the symptoms recurred with violence. The abdomen swelled, and the man died, after some hours, in extreme agony.

*Dissection of the Abdomen.* The peritoneal cavity and large intestines distended by gas. The small intestines, contracted, red, inflamed, covered with albuminous exudations and purulent flocculi, were thrust towards the pelvis. Their convolutions adhered intimately to each other, and to the omentum, as they approached the abdominal rings, particularly the right. Between them and the contiguous portions of the mesentery and omentum, were found, here and there, large collections of pus. A loop of ileum, detached from the mass, presented two contractions, distant about four inches from each other, and not wholly obliterating its canal. Probably the pressure, exercised by the ring on this portion of the intestine, during the strangulation of the left hernia, had produced this double contraction.

The adhesions which united the packet of intestine to the right abdominal ring and its circumference were cautiously destroyed; and I then saw that neither intestine nor omentum entered this orifice; but that it was completely closed by the adhesion of the above-mentioned parts, as well as by the occlusion of the hernial sac. The peritonæum, continued into the ring, and forming a funnel, of little depth, constituted clearly the origin of the sac; but it was obstructed. My finger, pushed into this funnel, as if to traverse the ring from within outwards, overcame the resistance, and penetrated, with violence, into the interior of the tumour.

On opening the latter, its whole length, there escaped about two ounces of a white, puriform, flaky liquid, somewhat ropy, and without smell; in fact, closely resembling the fluid contained in the intestinal collections. The parietes of the tumour were evidently continuous with the portions of peritonæum, which dipped in behind the ring. They were about four lines thick, white, firm, and very elastic. On removing the layers, I could distinguish no appearance of fibres, in the direction in which I cut. Its posterior surface lay on the vessels composing the spermatic cord. Its internal surface, smooth, but here and there inflamed, had a corroded appearance, and was superficially ulcerated, almost throughout.

*Reflections.* Ought the symptoms of strangulation to be ascribed to the left hernia, before reduction? And how far did the double contraction of the intestine composing this hernia, serve to keep up the symptoms after its reduction? Did compression of a part of the circumference of the intestine by the right inguinal ring, or rather by the contracted neck of the hernial sac, contribute to the symptoms of strangulation; and was the inflammation there originating, propagated, step by step, to the whole intestinal mass? Thirdly, Could a loop of intestine, engaged in the sac, have been so compressed as to suffer inflammation; and the reduction of the intestine not being perceived from the density and thickness of the sac, would that inflammation continue, and become diffused to the rest of the intestine? To decide positively to which of these causes the intestinal inflammation was attributable, would be difficult, or perhaps, impossible.

The operation was not only useless, but probably aggravated the condition of the patient: and the irritation which it produced on the ring and its vicinity, must have augmented that already existing behind it. And would not the general antiphlogistic treatment, modified according to the seat of the inflammation, have sufficed to operate its resolution, and allay, or completely remove the symptoms? The double intestinal contraction was not so complete as to obstruct the passage of the aliment, taken in moderation, through the canal. But the hope, inspired by a favourable change of the symptoms two days after the operation, was utterly destroyed by the imprudence of the patient. The indigestible food which he took, undoubtedly hurried him to the grave.

If the operator had known precisely the nature of the tumour, its removal might have been advantageous. Mistaken for strangulated hernia, and analogous to the hernial sac, in its volume, size, figure, and relations, this tumour did not sensibly alter in size from the developement of the symptoms till death. It was then principally, perhaps not exclusively, formed by the morbid hernial sac: for it is very possible that a loop of intestine, contained originally in the sac, had been reduced without the volume of the latter being diminished; when we consider the thickness, solidity, and above all, the elasticity of its parietes.

Did the tumour commence with the developement of the symptoms? or had it, for some time previously, existed; but, from its indolence, unperceived by the patient? In the first case, the inflammation of the sac irritated by the displaced parts and by the attempts at reduction, would have induced the thickening of its parietes: in the last, the secondary inflammation, determined by the same causes, would have increased the morbid change already existing. The dissection leaves no doubt as to the immediate seat, and nature of this tumour.

(We shall proceed with this interesting memoir in our next number. Ed.)

### PART IV.

### MEDICAL MISCELLANIES.

### THE PORTE FEUILLE, No. II.

OR,

DEPOT FOR DETACHED FACTS AND INTERESTING PHÆNOMENA, OCCURRING IN THE PRACTICE OF THE FRIENDS OF THE MEDICO-CHIRURGICAL JOURNAL.

"Ore trahit quodcunque potest atque addit acervo."

